# FI-CEUS: a solution to improve the diagnostic accuracy in MRI LI-RADS-indeterminate (LR-3/4) FLLs at risk for HCC

**DOI:** 10.3389/fonc.2023.1225116

**Published:** 2024-01-17

**Authors:** Qingjing Zeng, Sidong Xie, Xuqi He, Yuefei Guo, Yuxuan Wu, Na He, Lanxia Zhang, Xuan Yu, Rongqin Zheng, Kai Li

**Affiliations:** ^1^ Department of Ultrasound, The Third Affiliated Hospital of Sun Yat-sen University, Guangzhou, China; ^2^ Department of Radiology, The Third Affiliated Hospital of Sun Yat-sen University, Guangzhou, China; ^3^ Department of Ultrasound, The Third Affiliated Hospital of Sun Yat-sen University-Yuedong Hospital, Meizhou, China

**Keywords:** contrast-enhanced ultrasound, contrast-enhanced magnetic resonance imaging, fusion imaging, hepatocellular carcinoma, Liver Imaging Reporting and Data System

## Abstract

**Objective:**

To evaluate the diagnostic accuracy of fusion imaging contrast-enhanced ultrasound (FI-CEUS) of magnetic resonance imaging (MRI) LI-RADS-indeterminate (LR-3/4) and conventional ultrasound undetected focal liver lesions (FLLs) in patients at risk for hepatocellular carcinoma (HCC).

**Methods:**

Between February 2020 and July 2021, 71 FLLs in 63 patients were registered for diagnostic performance evaluation respectively for ultrasound-guided thermal ablation evaluation in this retrospective study. Diagnostic performance regarding FLLs was compared between FI-CEUS and contrast-enhanced MRI (CE-MRI).

**Results:**

For diagnostic performance evaluation, among 71 lesions in 63 patients, the diagnostic efficacy of FI-CEUS with LI-RADS was significantly higher than that of CE-MRI (*P* < 0.05) in both overall and hierarchical comparison (except for the group with lesion diameter ≥2 cm). For malignant lesions, the proportion of arterial phase hyperenhancement (APHE) and washout on FI-CEUS was higher than that on CE-MRI (*P* < 0.05).

**Conclusion:**

FI-CEUS has a high value in the precise qualitative diagnosis of small FLLs (<2 cm) of MRI LI-RADS-indeterminate diagnosis (LR-3/4) that are undetected by conventional ultrasound in patients at risk for HCC and can be a good supplementary CE-MRI diagnostic method for thermal ablation evaluation.

## 1 Introduction

Hepatocellular carcinoma (HCC) is the fifth most common cancer and the second leading cause of cancer death worldwide and usually occurs in patients with risk factors for HCC (1, 2). Non-invasive medical imaging, especially contrast-enhanced imaging [e.g., contrast-enhanced magnetic resonance imaging (CE-MRI], plays a crucial role in the qualitative diagnosis of HCC. Typical enhanced manifestations of HCC include arterial phase hyperenhancement followed by gradual washout in the portal venous and late phase (3–6). However, there are atypical MRI manifestations of liver nodules in patients with a high risk of HCC for which MRI diagnosis is challenging and affects treatment. The reasons include the changes in lesion artery and portal vein blood supply during the development of HCC, time deviation of MRI image acquisition, etc. (6–10). The American College of Radiology (ACR) Liver Imaging Reporting and Data System (LI-RADS) was initiated to standardize the classification of liver findings from high-risk patients according to lesion size and enhancement characteristics for grading from absolute benign to absolute HCC and has become a standardized evaluation method for enhanced imaging in the diagnosis of HCC (11–14). Such liver nodules with atypical MR manifestations in patients with a high risk of HCC are defined as LR-3/4, of which the diagnosis of whether these suspicious lesions are HCCs will affect the staging of HCC in patients and ultimately affect treatment decisions and prognosis before ultrasound-guided thermal ablation evaluation.

Over the years, contrast-enhanced ultrasound (CEUS) has been used as a first-line diagnostic method for HCC in Europe and Asia, and its diagnostic value in HCC has been widely accepted (15–23). For liver nodules with atypical MRI manifestations visible by conventional ultrasound (US), CEUS was used to supplement the diagnosis, which can improve accuracy in diagnosing such lesions (17, 24–26). However, for liver nodules invisible by conventional US, detection by routine CEUS examination would be difficult, ACR LI-RADS was not recommended for this type of lesions (13, 15), and there would be difficulties in diagnosis, ultrasound-guided puncture biopsy, and thermal ablation.

Fusion imaging (FI) techniques can help to precisely locate lesions with poor conspicuity on conventional US, permitting two-dimensional (2D) multiplanar reconstruction (MPR) images from CT or MRI to be displayed in the same plane as the US images by moving the US transducer (27–32). CEUS-MR fusion imaging combines the advantages of precise positioning of fusion imaging and qualitative diagnosis of CEUS, which may be a solution for the problem of detecting such MRI-indeterminate and conventional US-invisible liver lesions. To date, there is little relevant research literature.

The aim of our study was to evaluate diagnostic accuracy when applying FI-CEUS to MRI-indeterminate (LR-3/4) and conventional US-undetected focal liver lesions (FLLs) in patients at risk for HCC.

## 2 Materials and methods

Our retrospective study was approved by the institutional ethics committee (Ethical approval number: II2023-142-01), and the requirement of written informed consent was waived.

### 2.1 Study population

**Figure 1:**
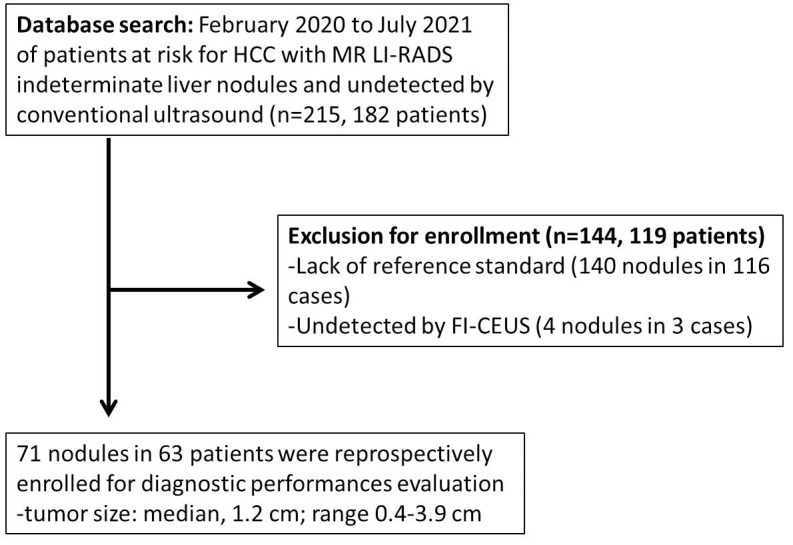
Flowchart of the study cohort. The following chart shows how the cohort was derived, giving numbers for included cases at each step.

Consecutive hepatic CEUS-MRI fusion imaging examinations performed at The Third Affiliated Hospital of Sun Yat-sen University between February 2020 and July 2021 were retrospectively evaluated. The images represented patients with a high risk of HCC who presented with untreated FLLs of MRI LI-RADS-indeterminate diagnosis (LR-3/4) that were undetected by conventional US and were planned to undergo thermal ablation treatment. The inclusion and exclusion criteria were as follows (**Figure 1**).

### 2.2 Inclusion criteria

1) Patients at high risk of HCC (aged 18–80 years), including cirrhosis and chronic hepatitis B and C.
2) CE-MRI showed FLLs, and the diagnosis was indeterminate by CE-MRI and undetectable by conventional US.
3) CEUS-MR fusion imaging examination was performed, and the interval between MRI and FI-CEUS was within 1 month. Moreover, MR and ultrasound can be successfully matched.
4) The lesion without treatment (TACE or immunotherapy, radiotherapy, etc.) before examination of the patient.

### 2.3 Exclusion criteria

1) Inability to obtain a reference standard, including without pathology results or no long-term follow-up (follow-up period of at least 1 year) results.
2) The lesion undetected by FI-CEUS.

### 2.4 Examination and image

#### 2.4.1 MRI examination

MRI studies were performed following standardized protocols on GE Optima MR360 1.5T, GE Discovery MR750 3.0T, GE SIGNA Architect 3.0T, and Siemens Prisma 3.0T scanners. MRI contrast agents included gadobenate dimeglumine, gadodiamide, gadobutrol, and gadoxetate disodium and were administered according to the manufacturer-recommended weight-based doses.

All patients underwent MRI examination procedures. Respiratory training was performed before examination, and end-expiratory scans were obtained. All patients underwent routine plain MRI (including axial T1WI, T2WI, and DWI) and contrast-enhanced scans (dynamic contrast-enhanced), and arterial, portal, and delayed phase images were acquired by injecting recommended dosage body mass of contrast medium with an equivalent volume of saline through the cubital vein at a 3-mm/s flow rate using a high-pressure injector and bolus injection of contrast material. Hepatobiliary phase images were acquired at 20 min.

#### 2.4.2 US and fusion imaging CEUS examination

US and fusion imaging CEUS examinations were performed using ultrasound systems (Esaote MyLab Class C, Esaote MyLab Twice, Esaote MyLab8) equipped with a two-dimensional convex array probe (CA541, C1-8) with a probe frequency ranging from 1 to 8 MHz. The image fusion system was configured and equipped with software for CEUS imaging technology. A low mechanical index (MI) parameter technique (CnTI imaging technique) was used, and the mechanical index was maintained below 0.20 to reduce the destruction of microbubbles.

#### 2.4.3 US operator requirements

Routine US examination was performed by a radiologist with more than 5 years of experience in liver diagnosis with CEUS. FI-CEUS examinations were performed by one radiologist with more than 5 years of experience in ultrasound FI examinations of the liver.

#### 2.4.4 Examination methods and image acquisition

A routine US scan imaging was used to judge whether the lesion was visible by two radiologists with more than 5 years of experience in diagnosis utilizing CEUS of the liver.

#### 2.4.5 FI-CEUS examinations

The process of FI-CEUS in these platforms was generally as follows: 1) MRI volume data in digital imaging and communication in medicine (DICOM) format were imported into the ultrasound unit through a USB hard drive; target lesions were marked on the MRI sequence images with clear display of target lesion. 2) The magnetic field generator was placed near the patient’s trunk, and the magnetic sensor was attached to the US transducer. 3) Real-time registration of MRI and US images was performed to achieve synchronous display in both modalities: 3a) For image registration, the sagittal part of the left portal vein was usually used for preliminary MRI and US registration. 3b) Reference points around lesions were also applied for registration. 4) After a good matching of MRI and US images, the lesions that were inconspicuous on B-mode US could be confirmed precisely. 5) CEUS-MR fusion examination: A bolus injection of 1.5 ml sulfur hexafluoride-filled microbubble contrast agent (SonoVue; Bracco, Milan, Italy) was injected via a 20-gauge catheter placed in the elbow vein. A 5-ml 0.9% sodium chloride solution was administered after injection of a contrast agent. After the contrast agent injection was completed, the imaging timer was started immediately. The region of interest (ROI), including the target lesion and the surrounding liver parenchyma, was continuously recorded during the first 120 s, followed by intermittent imaging until washout was confidently observed or liver parenchymal enhancement faded, which was typically after 5 min or more. The FI-CEUS was stored on the hard drive of the ultrasound system and copied to the portable hard drive for later evaluation.

### 2.5 Reference standard (only one reference standard of the two was required)

#### 2.5.1 Pathological diagnosis

Tumor histocytological examination was performed on resection samples obtained by laparoscopic-assisted or direct visual inspection or on needle biopsy samples obtained under FI guidance.

#### 2.5.2 Clinical follow-up diagnosis

According to the guidelines for the clinical diagnosis of HCC, follow-up examinations with CE-MRI or FI-CEUS were performed every 3–6 months, and the follow-up period was longer than 1 year for diagnostic evaluation according to the European Federation of Societies for Ultrasound in Medicine and Biology (EFSUMB) guidelines for the clinical diagnosis of HCC [5].

### 2.6 Data analysis

#### 2.6.1 CE-MRI diagnostic criteria

Two certified radiologists with more than 5 years of experience in liver CE-MRI who were blinded to the reference standard results and other imaging test results independently reviewed the CE-MRI examinations of the FLLs and assigned a category according to ACR MRI LI-RADS (version2018). The imaging criteria for the diagnosis of benign and malignant lesions are as follows: LR-5 category combined with LR-4 category FLLs was diagnosed as HCCs, while less than LR-4 category was diagnosed as benign lesions.

#### 2.6.2 FI-CEUS diagnostic criteria

Two certified radiologists with more than 5 years of experience in liver contrast-enhanced US who were blinded to the reference standard results and other imaging test results independently reviewed the contrast-enhanced US examinations of the liver nodules and assigned a category according to ACR CEUS LI-RADS (version 2017). The imaging criteria for the diagnosis of benign and malignant lesions are as follows: LR-5 category combined with LR-4 category FLLs was diagnosed as HCCs, while less than LR-4 category was diagnosed as benign lesions.

### 2.7 Statistical analysis

Data collation and statistical analysis were performed using Excel 2010 (Microsoft Office, Redmond, WA, USA) and MedCalc 12.7.0 (MedCalc, Ostend, Belgium). Descriptive parameters, including sex, age, liver background, history of hepatitis, AFP, and lesion size, were recorded. The detection rate was displayed as a percentage, and the comparison of detection rates was performed using Fisher’s exact test. According to the reference standard, diagnostic efficacy comparisons were performed by analyzing the areas under the ROC curves (AUCs) between MRI and FI-CEUS LI-RADS, where AUCs, sensitivity, and specificity were determined. *P*-values <0.05 were considered statistically significant.

## 3 Results

### 3.1 Study population

Between February 2020 and July 2021, 182 patients at risk for HCC with 215 nodules that were both CE-MRI LI-RADS-indeterminate (LR-3/4) and undetected by conventional US were retrospectively evaluated. Finally, 71 lesions in 63 patients were registered for the final analysis of diagnostic performances.

### 3.2 Diagnostic performance evaluation

#### 3.2.1 Characteristics of the patients and liver nodules

Seventy-one lesions confirmed by pathological or clinical follow-up in 63 patients at high risk of HCC (median age, 56 years; age range 29 to 77 years; gender distribution, 52 men and 11 women) were registered for the final analysis, consisting of 31 benign lesions (22 cirrhosis nodules, 4 hemangiomas, 1 adenoma, 3 inflammatory lesions, and 1 spontaneous portosystemic shunt) and 40 malignant lesions (HCCs). The median observation size (diameter) was 1.2 cm (range 0.4–3.9 cm).

The tally of the final CE-MRI LI-RADS scores was LR-3 (*n* = 64) and LR-4 (*n* = 7). The tally of the FI-CEUS LI-RADS scores was LR-3 (*n* = 26), LR-4 (*n* = 16), and LR-5 (*n* = 29). The characteristics of the patients and nodules are shown in **Table 1**.

**Table 1:** Characteristics of the enrolled patients and nodules for diagnostic performances.

Characteristics	Number
Gender (male/female)	52/11
Age (years)^a^	56 (29–77)
AFP (ng/ml)^a^	5.2 (1.2–1200.0)
Liver disease background	
Hepatitis B	60
Hepatitis C	1
Autoimmune cirrhosis	2
Tumor size (cm)^a^	1.2 (0.4–3.9)
Tumor location (segment):	
S2, S3, S4, S5, S6, S7, S8, S4/8, S5/6, S5/8, S6/7, S7/8	2/4/4/8/15/9/17/2/3/2/4/1
CE-MRI LI-RADS: L3/L4	64/7 26/16/29
FI-CEUS LI-RADS: L3/L4/L5	26/16/29
Diagnosis result:	
Benign (cirrhosis nodules/hemangioma/adenoma/inflammatory/spontaneous portosystemic shunt)	31(22/4/1/3/1)
Malignant (HCC)	40
Diagnosis method: pathological/clinical follow-up	56/15

AFP, a-fetoprotein; CE-MRI, contrast-enhanced magnetic resonance imaging; FI-CEUS, fusion imaging contrast-enhanced ultrasound; LI-RADS, Liver Imaging Reporting and Data System; HCC, hepatocellular carcinoma.

a Data are median (minimum, maximum).

#### 3.2.2 Overall comparison of FI-CEUS LI-RADS and CE-MRI LI-RADS categories

Regarding overall diagnostic performance, among 71 lesions in 63 patients, the AUC of FI-CEUS LI-RADS (0.802) was significantly higher than that of CEMRI LI-RADS (0.502) (*P* < 0.0001) (**Table 2**).

**Table 2:** Comparison of diagnostic performance values on FI-CEUS LI-RADS and CE-MRI LI-RADS categories.

			AUC	SE	Sensitivity (%)	95% CI	Specificity (%)	95% CI	*P*-values
Overall	*N* = 71	CE-MRI	0.502	0.0361	10	2.8–23.7	90.32	74.2–98.0	
		FI-CEUS	0.802	0.0520	87.5	73.2–95.8	67.74	48.6–83.3	*<0.0001*
Lesion size	<1 cm (*N* = 18)	CE-MRI	0.500	0	0	0.0–33.6	100	66.4–100.0	
		FI-CEUS	0.944	0.0556	88.89	51.8–99.7	100	66.4–100.0	*<0.0001*
	≥1 and <2 cm (*N* = 46)	CE-MRI	0.508	0.0372	7.14	0.9–23.5	94.44	72.7–99.9	
		FI-CEUS	0.809	0.0637	85.71	67.3–96.0	61.11	35.7–82.7	*0.0001*
	≥2 cm (*N* = 7)	CE-MRI	0.583	0.2200	66.67	9.4–99.2	50	6.8–93.2	
		FI-CEUS	0.750	0.1440	100	29.2–100.0	50	18.4–90.1	*0.5271*

CE-MRI, contrast-enhanced magnetic resonance imaging; FI-CEUS, fusion imaging contrast-enhanced ultrasound; AUC, area under the curve; SE, standard deviation; CI, confidence interval; N, number.

#### 3.2.3 Comparison of FI-CEUS LI-RADS and CE-MRI LI-RADS categories according to lesion size

For lesions <1 cm, the AUC of FI-CEUS (0.944) was significantly higher than that of CE-MRI (0.500) (*P* < 0.0001). For lesions ≥1 cm and <2 cm, the AUC of FI-CEUS (0.809) was significantly higher than that of CE-MRI (0.508) (*P* = 0.0001). For lesions ≥2 cm, there was no significant difference between the AUC of FI-CEUS (0.583) and that of CE-MRI (0.750) (*P* = 0.5271). A comparison of diagnostic performance values on FI-CEUS LI-RADS and CE-MRI LI-RADS is shown in **Table 2**.

#### 3.2.4 Comparison of FI-CEUS LI-RADS and CE-MRI LI-RADS categories according to MRI LI-RADS

For CE-MRI LR-3 lesions, the AUC of FI-CEUS (0.806) was significantly higher than that of CE-MRI (0.500) (*P* < 0.0001). For CE-MRI LR-4 lesions, the AUC of FI-CEUS (0.833) was significantly higher than that of CE-MRI (0.500) (*P* < 0.05) (**Table 3**).

**Table 3:** Comparison of FI-CEUS LI-RADS and CE-MRI LI-RADS categories according to MRI-enhanced pattern and MRI LI-RADS.

		AUC	SE	Sensitivity (%)	95% CI	Specificity (%)	95% CI	P-values
CE-MRI LR-3 (*N* = 64)	CE-MRI	0.500	0	0	0.00–9.70	100	87.70–100.0	
	FI-CEUS	0.806	0.0546	86.11	70.50–95.30	71.43	51.30–86.80	*<0.0001*
CE-MRI LR-4 (*N* = 7)	CE-MRI	0.500	0	0	0.00–60.20	100	29.20–100.0	
	FI-CEUS	0.833	0.167	100	39.80–100.0	66.67	9.40–99.20	*0.0455*

LR, Liver Imaging Reporting and Data System; CE-MRI, contrast-enhanced magnetic resonance imaging; FI-CEUS, fusion imaging contrast-enhanced ultrasound; AUC, area under the curve; SE, standard deviation; CI, confidence interval; N, number.

Among the 36 nodules confirmed to be malignant in CE-MRI LR-3, 11 nodules were upgraded to LR-4 by FI-CEUS, and 20 nodules were upgraded to LR-5 by FI-CEUS. Among the four nodules confirmed to be malignant in CE-MRI LR-4, all four nodules were upgraded to LR-5 by FI-CEUS (**Table 4**).

**Table 4:** Comparison of LI-RADS categories between CE-MRI and FI-CEUS in benign and malignant lesions.

	Malignant lesions (*N* = 40)			Benign lesions (*N* = 31)		
	FI-CEUS LR-3	FI-CEUS LR-4	FI-CEUS LR-5	FI-CEUS LR-3	FI-CEUS LR-4	FI-CEUS LR-5
CE-MRI LR-3 (*N* = 64)	5	11	20	20	4	4
CE-MRI LR-4 (*N* = 7)	0	0	4	1	1	1

LR, Liver Imaging Reporting and Data System; CE-MRI, contrast-enhanced magnetic resonance imaging; FI-CEUS, fusion imaging contrast-enhanced ultrasound; N, number.

#### 3.2.5 Comparison of FI-CEUS LI-RADS and CE-MRI LI-RADS categories according to MRI contrast agents

The main MR contrast agents in this study were gadoxemic disodium (24 cases with 28 lesions) and gadobenate dimeglumine (37 cases with 41 lesions), while there was one case for each of the other two contrast agents. According to the stratified analysis of different MR contrast agents, for atypical cases using a conventional contrast agent (gadobenate dimeglumine), the diagnostic efficacy (AUC) of FI-CEUS LI-RADS was better than that of MR LI-RADS (0.797 vs. 0.534, *P* = 0.0036), while for atypical cases using a hepatobiliary-specific contrast agent (gadoxemic disodium), the diagnostic efficacy (AUC) of FI-CEUS LI-RADS was also better than that of MR LI-RADS (0.792 vs. 0.521, *P* = 0.0058).

#### 3.2.6 Comparison of enhanced patterns between MRI and FI-CEUS in benign and malignant lesions

**Figure 2:**
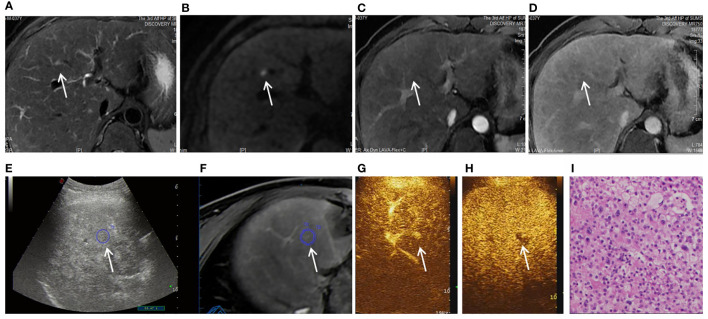
Images shown a 1.1 cm APNHE nodule in liver segment 8 in a 37-year-old man with chronic hepatitis B. **(A)** A high-signal nodule (arrow) was demonstrated in liver segment 8 on the MRI T2WI sequence. **(B)** High signal (arrow) was shown on the DWI sequence. **(C)** In the arterial phase, no hyperenhancement (arrow) was observed on CEMRI. **(D)** Nonperipheral washout (arrow) was observed in the late phase and was categorized as MRI LR-3. **(E, F)** After fusion imaging (**E**, ultrasound image; **F**, real-time synchronous MR image) navigation, the S8 nodule was not displayed by conventional grayscale US on the background of liver cirrhosis and fatty liver. **(G)** FI-CEUS showed hyperenhancement in the arterial phase compared with the surrounding liver parenchyma. **(H)** Mild washout (white arrow) was observed in the late phase and was categorized as FI-CEUS LR-5. **(I)** The patient underwent FI-CEUS-guided biopsy before microwave ablation, and the lesion was proven to be HCC at pathologic analysis.

**Figure 3:**
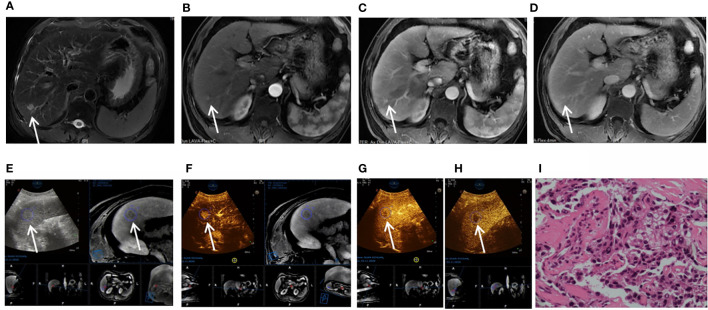
Images shown a 1.8 cm APHE nodule without washout in the venous and late phases in liver segment 6/7 in a 37-year-old man with chronic hepatitis B. **(A)** A high-signal nodule (arrow) was demonstrated in liver segment 6/7 on MR T2WI sequence. **(B)** In the arterial phase, hyperenhancement (arrow) was shown on CEMR. **(C)** Hyperenhancement (arrow) was observed in the venous phase. **(D)** Hyperenhancement (arrow) was observed in the late phase and was categorized as MRI LR-3. **(E)** After fusion imaging (ultrasound image on the left; real-time synchronous MR image on the right) navigation, the S6/7 nodule (arrow) was not displayed by conventional grayscale ultrasound on the background of liver cirrhosis. **(F)** FI-CEUS showed hyperenhancement (arrow) in the arterial phase compared with the surrounding liver parenchyma. **(G, H)** Mild washout (white arrow) was shown in the venous phase **(G)** and late phase **(H)** and was categorized as CEUS LR-5. **(I)** The patient underwent FI-CEUS-guided biopsy before microwave ablation, and the lesion was proven to be moderately differentiated HCC at pathologic analysis.

Among the 40 malignant nodules, the ratio of hyperenhancement ones in the arterial phase was significantly higher for FI-CEUS (87.50%, 35/40) than for CE-MRI (42.50%, 17/40) (*P* = 0.0001), while the ratio of the washout ones showed more lesions detectable in FI-CEUS (85.00%, 34/40) than in CE-MRI (55.00%, 22/40) (*P* = 0.0075) (**Figures 2**, **3**).

For the other 31 benign nodules, the ratio of hyperenhancement ones was not different between FI-CEUS (45.16%, 14/31) and CE-MRI (45.16%, 14/31) (*P* = 1.0000). The ratio of the washout ones showed no difference between FI-CEUS (21.58%, 7/31) and CE-MRI (25.81%, 8/31) (*P* = 1.0000) (**Table 5**).

**Table 5:** Comparison of enhanced patterns between CE-MRI and FI-CEUS in benign and malignant lesions.

	Malignant lesions (*N* = 40)		Benign lesions (*N* = 31)	
	Arterial phase hyperenhancement	Delayed phase washout	Arterial phase hyperenhancement	Delayed phase washout
CE-MRI	(17/40)	(22/40)	14/31	8/31
FI-CEUS	(35/40)	(34/40)	14/31	7/31
*P*	0.0001	0.0075	1.0000	1.0000

CE-MRI, contrast-enhanced magnetic resonance imaging; FI-CEUS, fusion imaging contrast-enhanced ultrasound; N, number.

## 4 Discussion

Early and accurate diagnosis of FLLs in patients at high risk of HCC has a significant impact on treatment and prognosis, especially for nodules with a diameter ≤2 cm (2–5). As the main non-invasive medical imaging diagnostic method of HCC, contrast-enhanced MR plays a more important role in the detection and qualitative diagnosis of HCC (6–10). However, due to many influencing factors, including the complexity of liver dual blood supply, changes in lesion artery and portal vein blood supply during the development of HCC, different histological stages of HCC, and time deviation of CE-MRI image acquisition, MRI manifestations of some HCCs are atypical and classified as intermediate probability (LR-3) or probable (LR-4) for HCC by ACR LI-RADS criteria, necessitating further evaluation, close follow-up, and sometimes early intervention (17, 24, 25).

Compared with conventional US (which is affected by many influencing factors such as echo, location, size, and liver background), MR-CEUS fusion imaging can allow accurate location of the FLLs through real-time MRI navigation with CEUS high-contrast examination for lesion recognition. CEUS can detect the whole-process real-time dynamic blood perfusion of liver nodules and has been used to supplement the diagnosis of liver nodules with unclear CE-MRI diagnosis that were visible by conventional US, which could improve diagnostic accuracy regarding such lesions (17, 24–26). However, liver nodules invisible by conventional US, which could account for 24% of all HCCs (28), are difficult to locate precisely and challenging for routine CEUS. The imaging characteristics of HCC also affect the detection of CEUS. For HCC, especially in the early stage (diameter ≤ 2 cm), the arterial phase is short, and the washout in the delayed phase is not obvious (often shown as mild washout) and possibly not found by whole liver scanning. MR-CEUS fusion imaging combined with the dual advantages of accurate lesion location and observation of the characteristics of whole-process microcirculation perfusion is expected to solve the precise positioning and diagnostic problem of MR-indeterminate and US-undetectable liver nodules. The findings in the literature (28) suggest that FI-US (detection rate: 98%, 85/87) can significantly improve the detection rate of HCC compared with conventional US (detection rate: 76%, 66/87).

ACR CEUS LI-RADS is not recommended to be used for FLLs that are not clearly displayed by conventional ultrasound (13, 15). The reason may be that these nodules are difficult to locate accurately for CEUS, which affects the entire observation process of CEUS and makes it difficult to obtain accurate LI-RADS categories. Furthermore, FI-CEUS achieves precise localization of lesions through MR navigation, which can obtain clear and complete blood flow perfusion features, ensuring the accuracy of LI-RADS. The results of our study showed that 71 lesions were accurately located through fusion imaging, and the LI-RADS score was obtained through clear contrast-enhanced ultrasound dynamic images throughout the entire process, demonstrating good diagnostic accuracy. Our study showed that MR-CEUS fusion imaging can improve accuracy in diagnosing CE-MRI LI-RADS-indeterminate liver nodules (LR-3/4) in patients at risk for HCC because of accurate positioning and qualitative diagnosis. In the overall case comparison, the diagnostic efficiency of FI-CEUS LI-RADS (0.802) was higher than that of CE-MRI (0.508) (*P* < 0.0001). FI-CEUS with accurate localization by fusion navigation and with real-time dynamic observation, which allows observation of the whole-process perfusion characteristics of lesions, achieved better appreciation in arterial hyperenhancement and/or washout. In our patients, for malignant lesions (*n* = 40), the ratio of hyperenhancement ones in the arterial phase was significantly higher for FI-CEUS (87.50%, 35/40) than CE-MRI (42.50%, 17/40) (*P* = 0.0001), while the ratio of the washout ones showed more lesions detectable in FI-CEUS (85.00%, 34/40) than in CE-MRI (55.00%, 22/40) (*P* = 0.0075). For benign lesions, regarding both arterial phase hyperenhancement and delayed phase washout, there were no differences between FI-CEUS and CE-MRI. Thus, FI-CEUS upgraded LI-RADS categories by observing more hyperenhancement in the arterial phase and washout in the delayed phase, thereby improving the accuracy of malignant nodule diagnosis. This may be one of the reasons why FI-CEUS LI-RADS had higher diagnostic efficiency than CE-MRI for such FLLs. In contrast to the intermittent image acquisition of CE-MRI, the whole-process observation of CEUS reduces the lack of high enhancement observation in the arterial phase. To address the problem of washout, in contrast to CE-MRI, a small molecule contrast agent is used, which easily penetrates the extracellular space of the tumor, resulting in prolonged enhancement of the tumor, while the ultrasound contrast agent is a pure “blood pool” agent that remains in the vessels during the vascular phases and allows more accurate observation of the washout (24).

According to the hierarchical analysis of lesion size, the diagnostic efficiency of FI-CEUS was higher than that of CE-MRI in diagnosing lesions of diameter <2 cm (*P* < 0.0001), while there was no significant difference in the diagnosis of larger lesions (lesion diameter ≥2 cm) (*P* = 0.5271). These results show that FI-CEUS LI-RADS was more valuable in the diagnosis of small (<2 cm) CE-MRI LI-RADS-indeterminate (LR-3/4) lesions. In contrast to previous studies on lesions visible by conventional ultrasound (17, 24–26), our study involved a comparative study on liver nodules with unclear display by conventional ultrasound and unclear CE-MRI diagnosis in patients at risk for HCC. The results confirmed that FI-CEUS had high diagnostic value in assessing such lesions.

In this study, according to the stratified analysis of different MR contrast agents, for atypical cases using a conventional contrast agent (gadobenate dimeglumine), the diagnostic efficacy (AUC) of FI-CEUS LI-RADS was better than that of MR LI-RADS (0.797 vs. 0.534, *P* = 0.0036), while for atypical cases using a hepatobiliary-specific contrast agent (gadoxemic disodium), the diagnostic efficacy (AUC) of FI-CEUS LI-RADS was also better than that of MR LI-RADS (0.792 vs. 0.521, *P* = 0.0058). The statistical results showed that whether it was atypical lesions with conventional MR contrast agents or with MR hepatobiliary-specific contrast agents, FI-CEUS LI-RADS had supplementary diagnostic value.

FI-CEUS also has shortcomings. Its influencing factors include FLL size; location, such as adjacency to the diaphragm with lung gas occlusion; and operator experience. In our study, FI-CEUS failed to detect four FLLs in three cases because of the small size of the lesions (0.4–0.6 cm). Therefore, we should fully understand the scope of the application of FI-CEUS to better solve clinical problems.

There were several limitations to our study. First, this study was a retrospective analysis, and prospective research is needed in the later stage to make the research more comprehensive and reliable. Second, some cases did not receive a pathological diagnosis and were diagnosed by long-term follow-up, which may affect the statistical results. Thirdly, based on the inclusion criteria bias, all selected lesions were cases with indeterminate CE-MRI diagnosis. Therefore, this study cannot prove that FI-CEUS was superior to CE-MRI for FLL diagnosis but only served as an effective supplement to CE-MRI in special clinical scenarios. Finally, our study population was relatively small and was pooled from a single medical center, thus precluding the generalization of our study results.

In conclusion, FI-CEUS LI-RADS has a high value in the precise detection and qualitative diagnosis of small FLLs (<2 cm) with CE-MRI LI-RADS-indeterminate diagnosis (LR-3/4) that are undetected by conventional US in patients at risk for HCC, and it can be a good supplementary MR diagnostic method.

## Data availability statement

The original contributions presented in the study are included in the article/supplementary material. Further inquiries can be directed to the corresponding author.

## Ethics statement

The studies involving humans were approved by Ethics Committee of the Third Affiliated Hospital of Sun Yat-sen University. The studies were conducted in accordance with the local legislation and institutional requirements. The participants provided their written informed consent to participate in this study.

## Author contributions

QZ, SX, and XH contributed equally to this paper and should be considered co-first authors for their equal contribution in study design, clinical data collection, data analysis and manuscript drafting and revision. YG, YW, and NH collected the data. LZ and XY analyzed the data. RZ and KL supervised the study. All authors contributed to the article and approved the submitted version.
